# Complications and recurrence risks after endoscopic resection of digestive neuroendocrine tumors: a retrospective study

**DOI:** 10.1186/s13023-025-03992-x

**Published:** 2025-09-02

**Authors:** Yuan Si, HongZhi Wu, Chao Wang, ZongXian Niu, Bo Wang, XianHui Zhang

**Affiliations:** 1 Endoscopic Center, XingTai People’s Hospital, Xingtai City, 054001 China; 2 Department of Respiratory Medicine, XingTai People’s Hospital, Xingtai City, 054001 China; 3 Department of Pathology, XingTai People’s Hospital, Xingtai City, 054001 China; 4 Department of CT, XingTai People’s Hospital, No. 16, Hongxing Street, Xingtai City, 054001 Hebei Province China

**Keywords:** Endoscopic submucosal dissection, Endoscopic mucosal resection, Endoscopic procedural complications, Gastrointestinal tract neoplasia, Neuroendocrine carcinoma, Neuroendocrine tumors of the digestive system

## Abstract

**Background:**

Peri-and postoperative complications and recurrences are associated with the endoscopic surgical procedures for neuroendocrine tumors of the digestive system. This study aimed to evaluate the long-term outcomes and safety of endoscopic submucosal dissection and mucosal resection for neuroendocrine tumors in the digestive system.

**Methods:**

In a retrospective cohort study, variables of minimally invasive endoscopic treatments and follow-up recurrences of 100 males and females with neuroendocrine tumors of gastric, duodenal, and rectal lesions were collected from records and analyzed. The curative resection criteria were followed the European Society of Gastrointestinal Endoscopy (ESGE) guidelines. Endoscopic ultrasound (EUS) and/or biopsy with histological assessment) routinely performed on all lesions included in this study for lesion confirmation prior to endoscopic resection.

**Results:**

Tumor size from 6 to 11.3 mm and endoscopic surgery procedure time from 6 to 13 min were reported. Forty-nine, 44, and seven lesions were located in the gastric, rectal, and duodenal regions, respectively. Six (6%), 4 (4%), 16 (16%), and 5 (5%) patients reported bleeding, perforation, nausea, and vomiting, respectively, due to the surgical procedure(s). Five patients (5%) underwent recurrent endoscopic surgery. Local recurrences occurred in three (3%; two (2%) of gastric lesions and one (1%) of duodenal lesions; all grade 1) patients, and distal metastases occurred in two (2%) patients. None of the patients died during the follow-up period. Before surgery, grade 2 (*p* = 0.049), tumor size ≥ 9.5 mm (*p* = 0.041), and gastric tract and rectal lesions (*p* = 0.021) were associated with local and/or distal metastases.

**Conclusions:**

The prevalence of neuroendocrine tumors is high in the stomach and rectum, endoscopic resection may be safe, and high-grade tumors may be associated with a high risk of recurrence.

**Supplementary Information:**

The online version contains supplementary material available at 10.1186/s13023-025-03992-x.

## Background

Neuroendocrine tumors are rare gastrointestinal tract neoplasias [1], with 2 patients per 1,000,000 people per year [2]. However, these types of tumors are not limited to the gastrointestinal tract. Such paragangliomas can also be found in the neck region [2]. Gastrointestinal tract neuroendocrine tumors can be detected using endoscopic measurements, even at the initial stages, especially in the stomach, duodenum, and rectum [3]. Neuroendocrine tumors are classified into (1) neuroendocrine tumors (divided into G1/G2/G3 according to the KI-67 index), (2) neuroendocrine carcinoma (it is poorly differentiated than grade G3 tumors) [4]. Therefore, G3 is not a poorly differentiated neuroendocrine tumor and not a neuroendocrine carcinoma [5]. Tumor grade is a prognostic factor for tumor size and lymph node metastasis, which is correctly defined after surgery [6]. Therefore, the Chinese National Clinical Practice Guidelines recommend endoscopic resection for early neuroendocrine tumors of the digestive system in high-risk areas [7]. The most recent guidelines published by a learned society (French) concerning the management of localized and metastatic digestive neuroendocrine tumors [8] recommended endoscopic resection for 10 mm or less tumors and showed no factors predictive of metastases.

Several studies and meta-analyses [1, 9, 10, 11, 12] reported the safety and effectiveness of endoscopic resection for neuroendocrine tumors of the digestive system. However, there is a lack of several parameters, such as long-term outcome evaluations and evaluations of techniques adopted based on the safety and effectiveness of surgeries [1]. Endoscopic submucosal dissection and endoscopic mucosal resection are the most commonly used surgical methods for neuroendocrine tumors of the digestive system [13].

This study aims to evaluate the long-term outcomes and safety of endoscopic submucosal dissection and endoscopic mucosal resection for neuroendocrine tumors in the digestive system during a median 37 months (minimum 19 months to maximum 60 months) of follow-up of patients who underwent endoscopic surgeries in the Southwestern Hebei region of China.

## Methods

### Design

Retrospective collection of anonymized medical records.

### Data sources

Hospital records of the Xingtai People’s Hospital, Xingtai, China.

### Study population

**Fig. 1:**
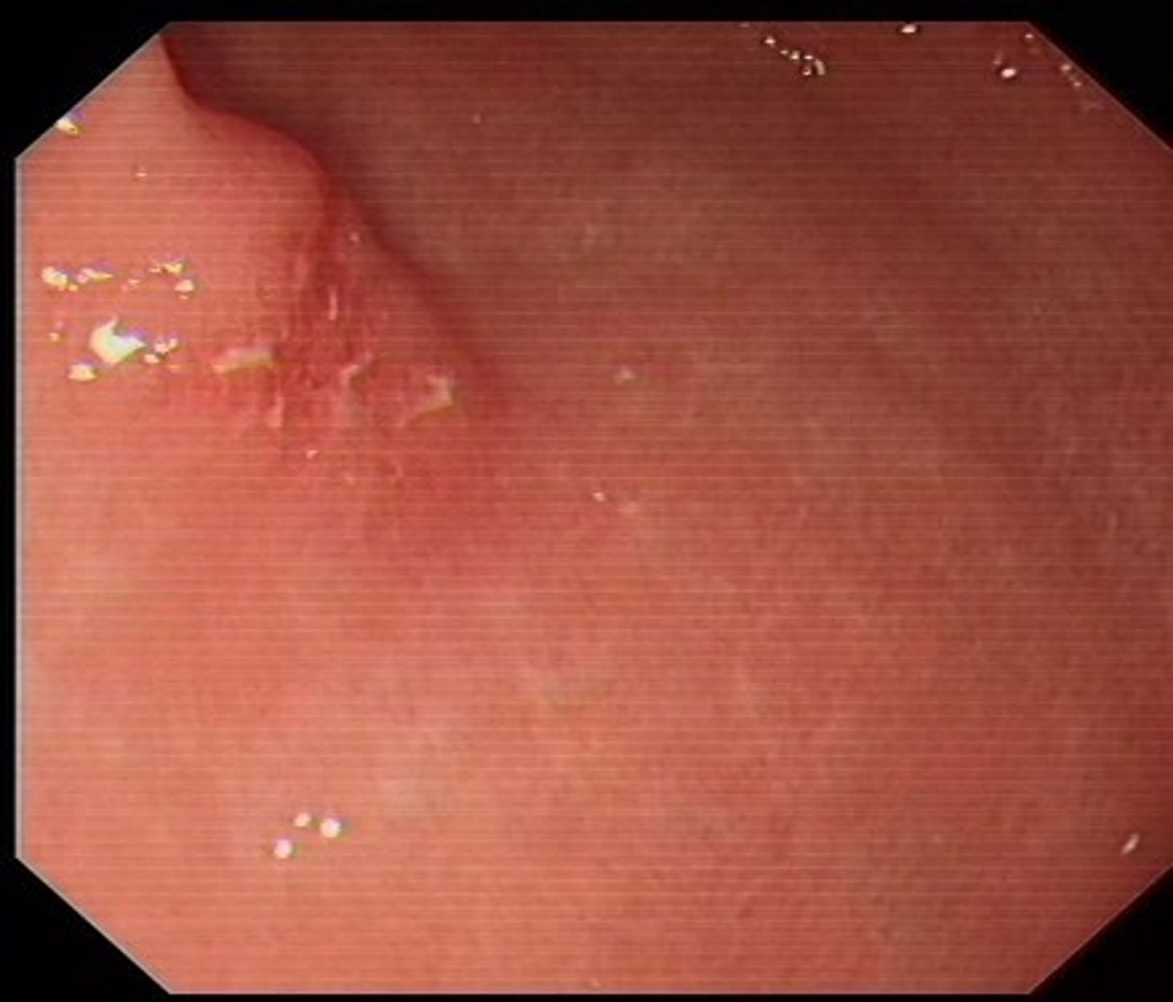
Gastric lesion before surgery

**Fig. 2:**
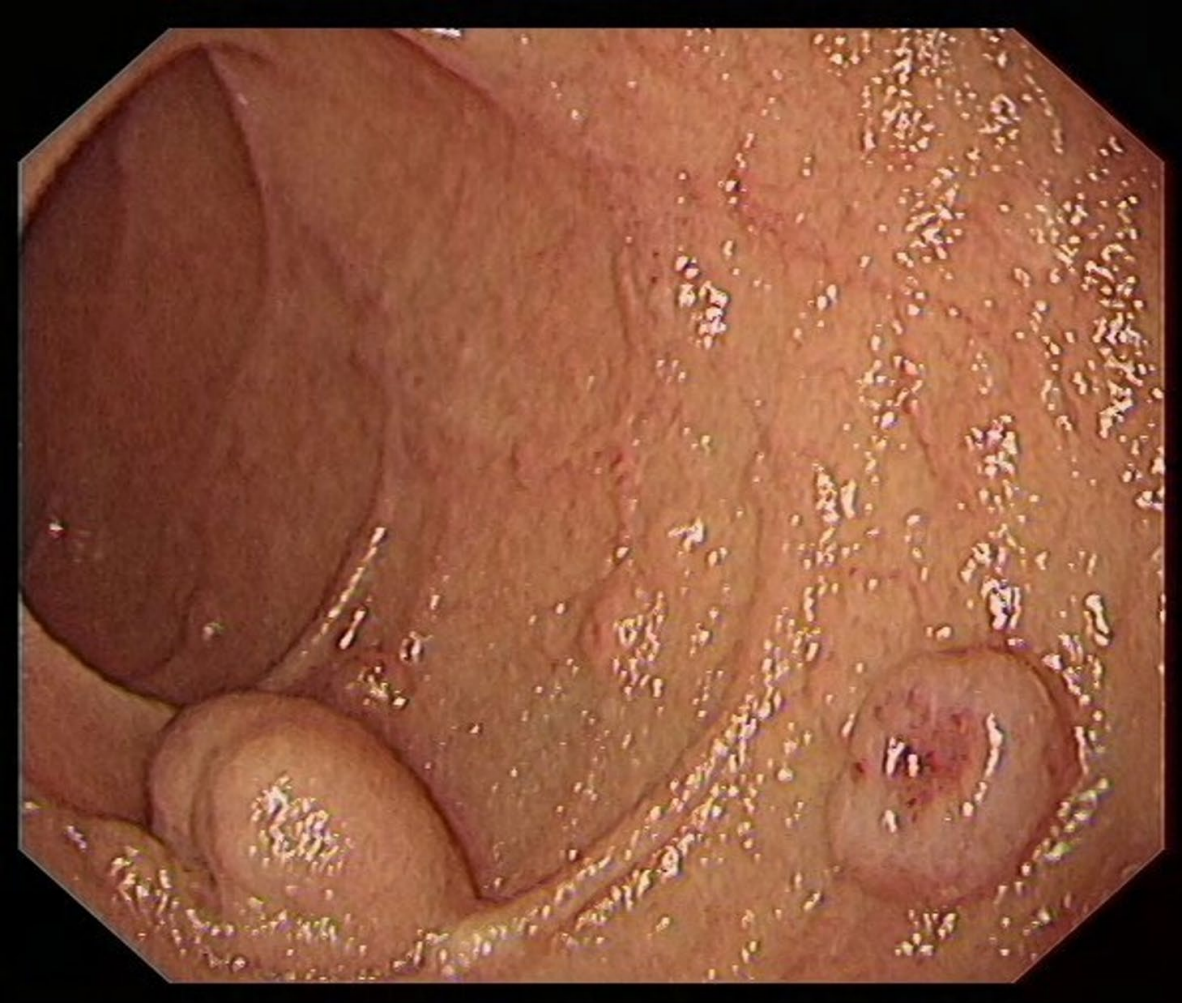
Duodenal lesion before surgery

**Fig. 3:**
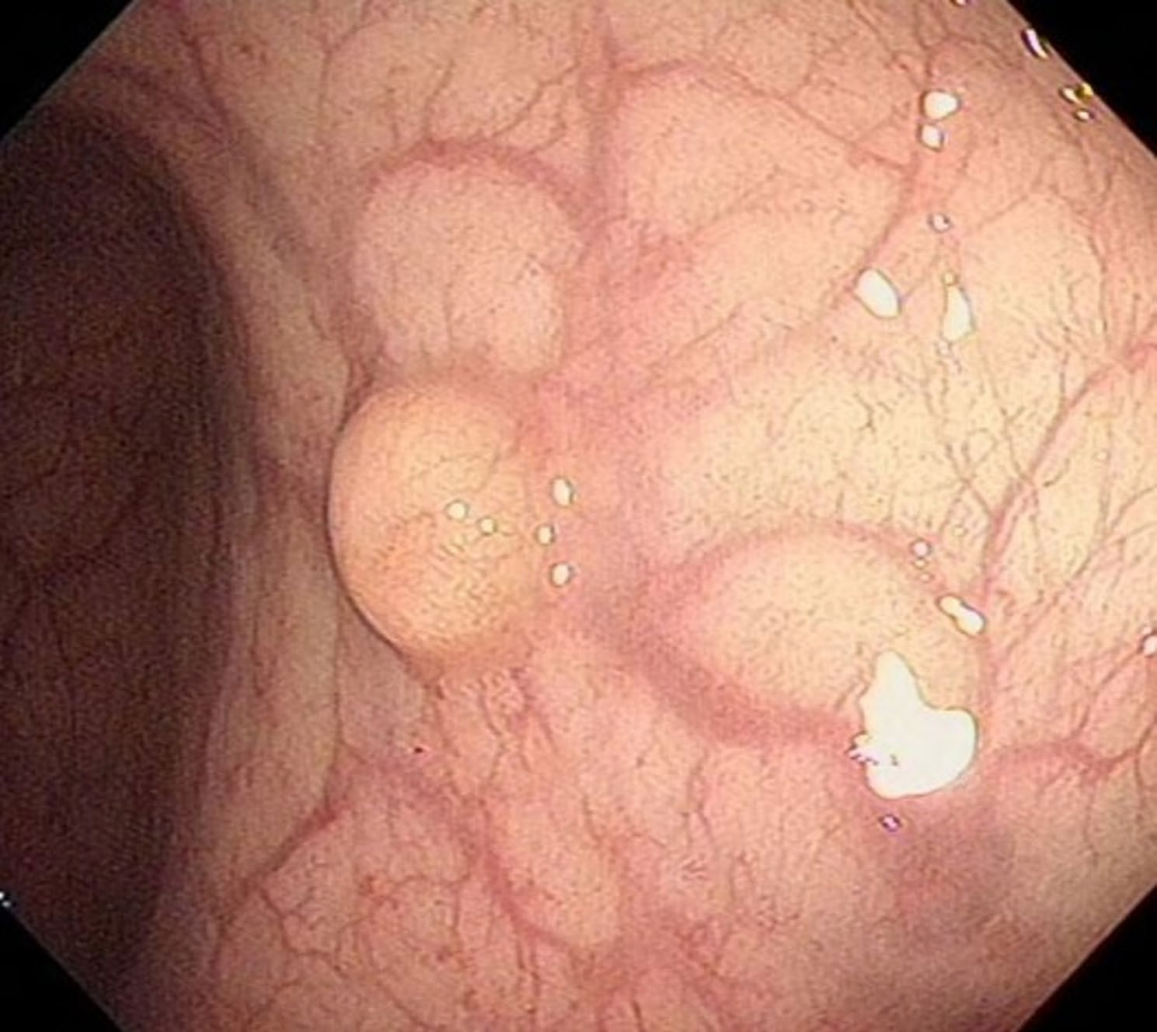
Rectal lesion before surgery

From January 2020 to December 2022 (the data collection period) study subjects were selected to collect 100 patients with neuroendocrine tumors of the digestive system (stomach (Fig. 1), duodenum (Fig. 2), or rectum (Fig. 3) who had received minimally invasive endoscopic treatments in the institute (a referral center for neuroendocrine tumors of the digestive system). A median of 37 months (range, 19–60 months) of follow-up data, including recurrences, were included in the study. All the enrolled patients were consecutive. Cases with a high risk of metastasis in the pathological diagnosis after endoscopic treatment, such as grade 2, positive lymphovascular invasion, and large lesions, were included in the analyses. Patients were eligible if endoscopic resection was performed on the lesion and diagnosed as R0 resection. G3 grade neuroendocrine tumors grow rapidly and metastasize at an early stage, which is not an indication for endoscopic surgery [14]; the G3 grade was removed from the evaluation criteria, and only the G1 and G2 grades suitable for endoscopic resection were retained in the analyses. Cases in which surgery was performed after endoscopic treatment were excluded from the analyses. The patients were consecutively selected.

### Endoscopic procedure

**Fig. 4:**
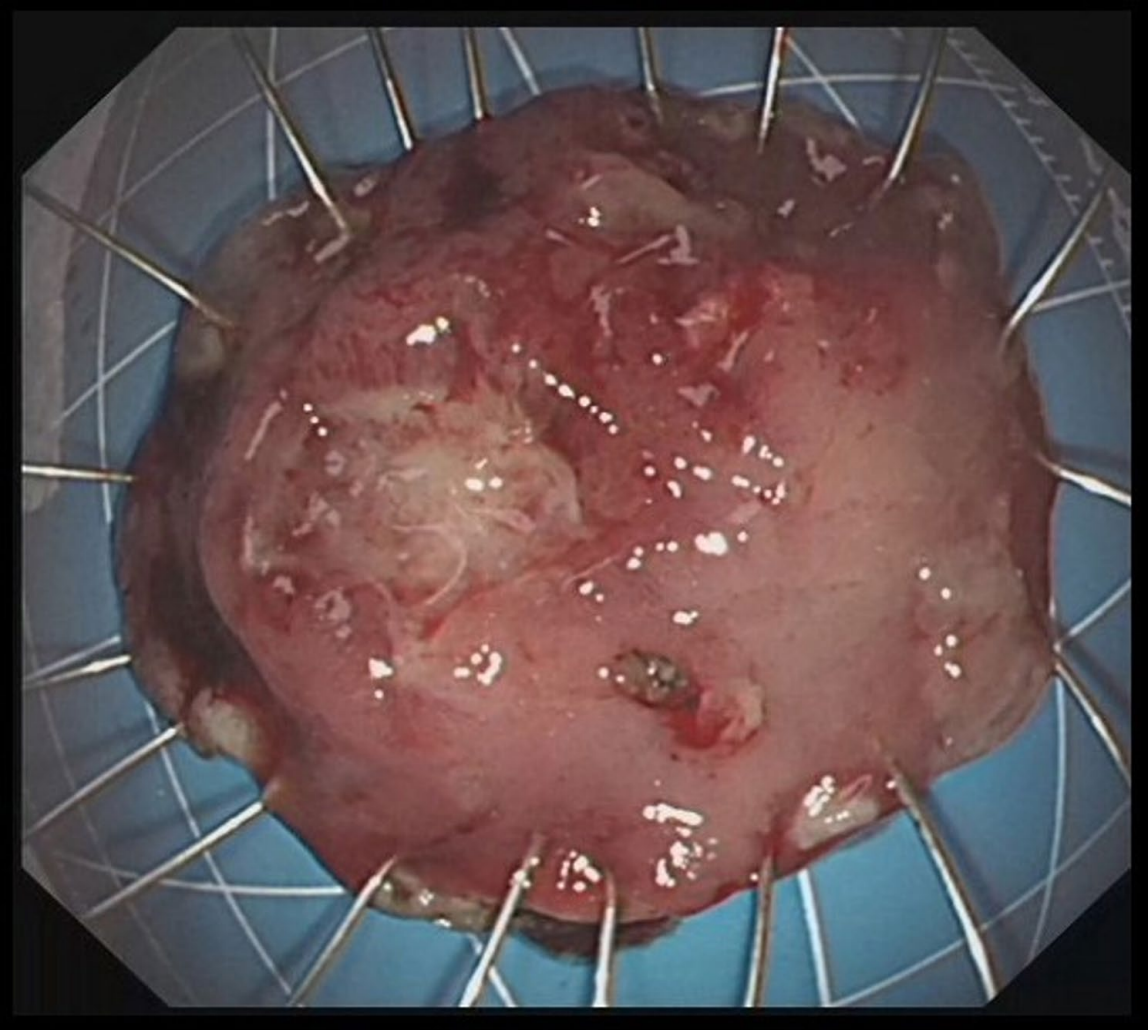
Endoscopic mucosal resection of gastric lesions

**Fig. 5:**
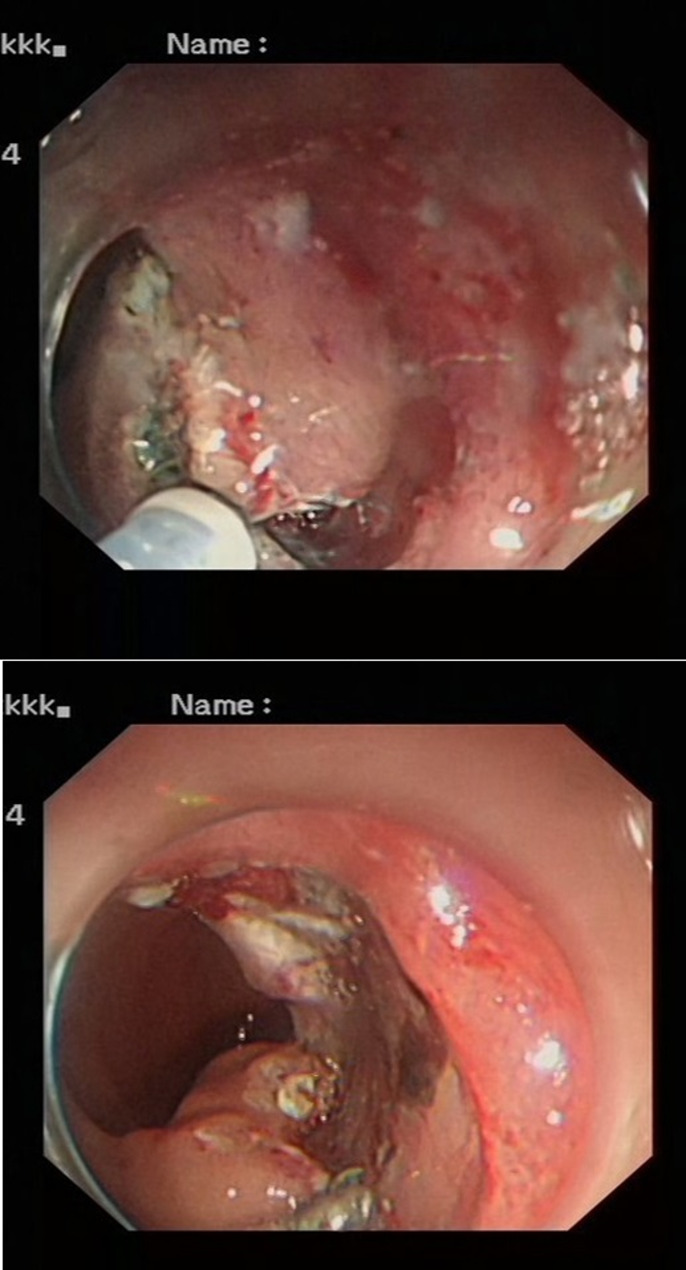
Endoscopic mucosal resection of duodenal lesions. A hybrid ESD-EMR technique

**Fig. 6:**
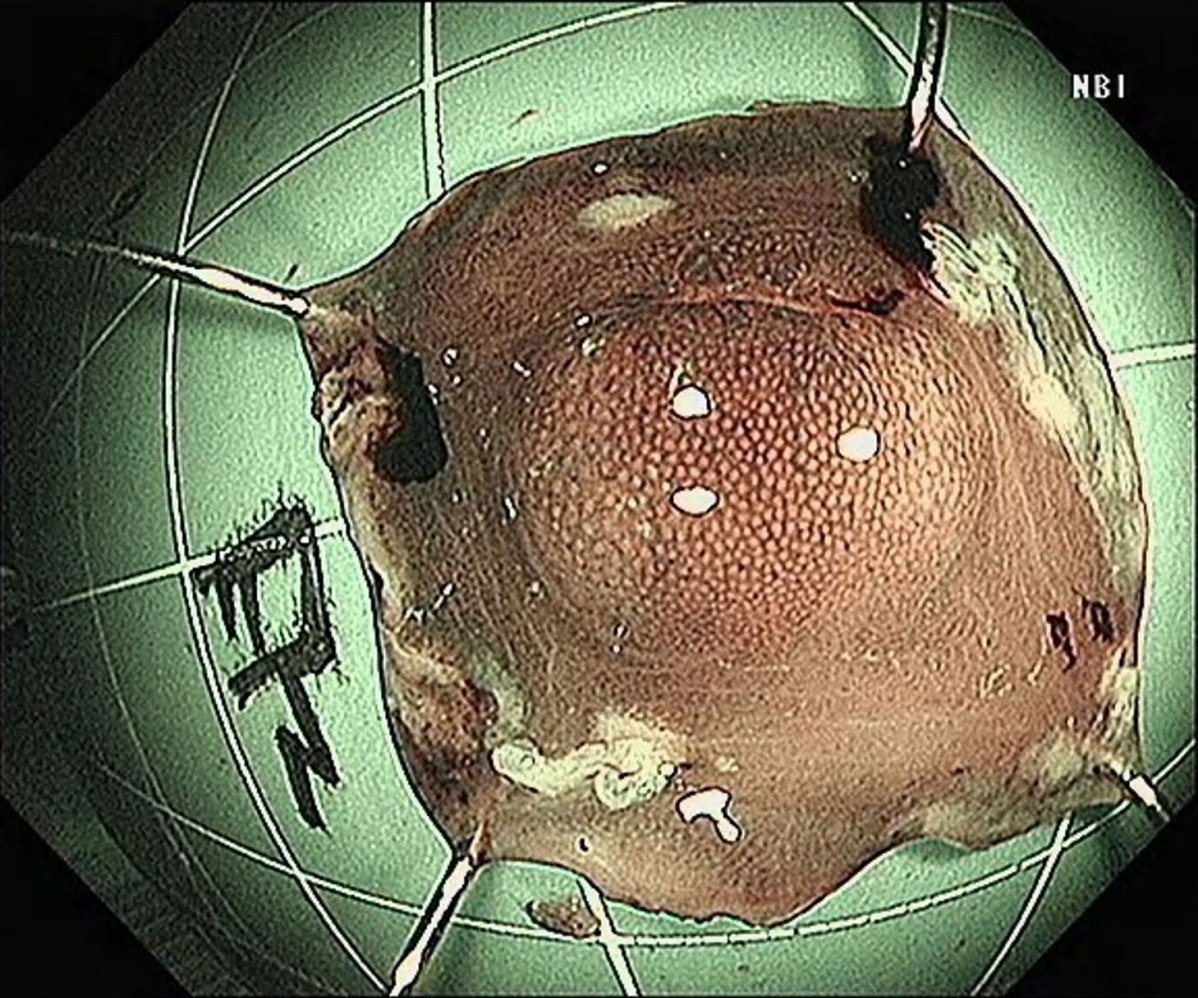
Endoscopic mucosal resection of rectal lesions

**Fig. 7:**
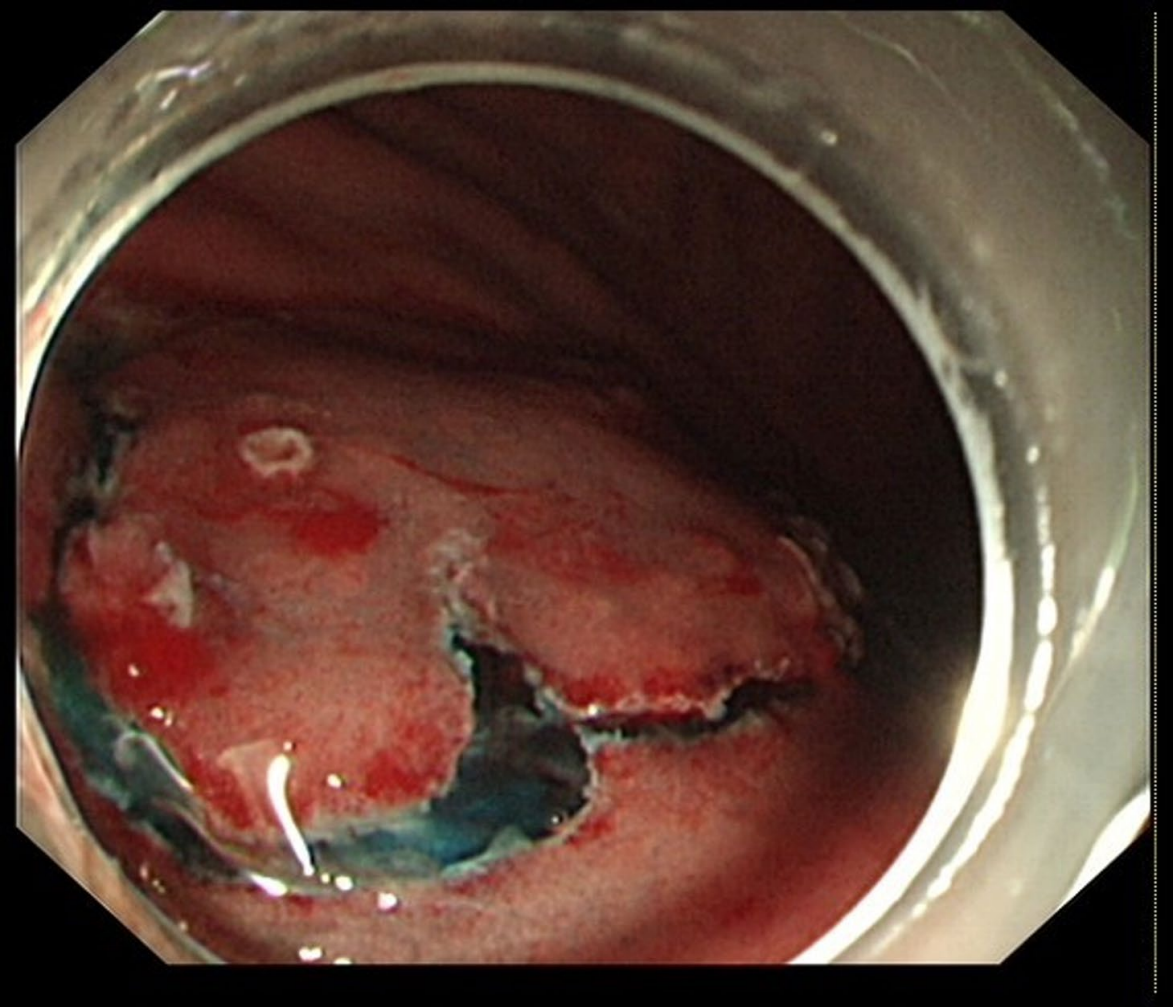
Endoscopic submucosal dissection of gastric lesions

**Fig. 8:**
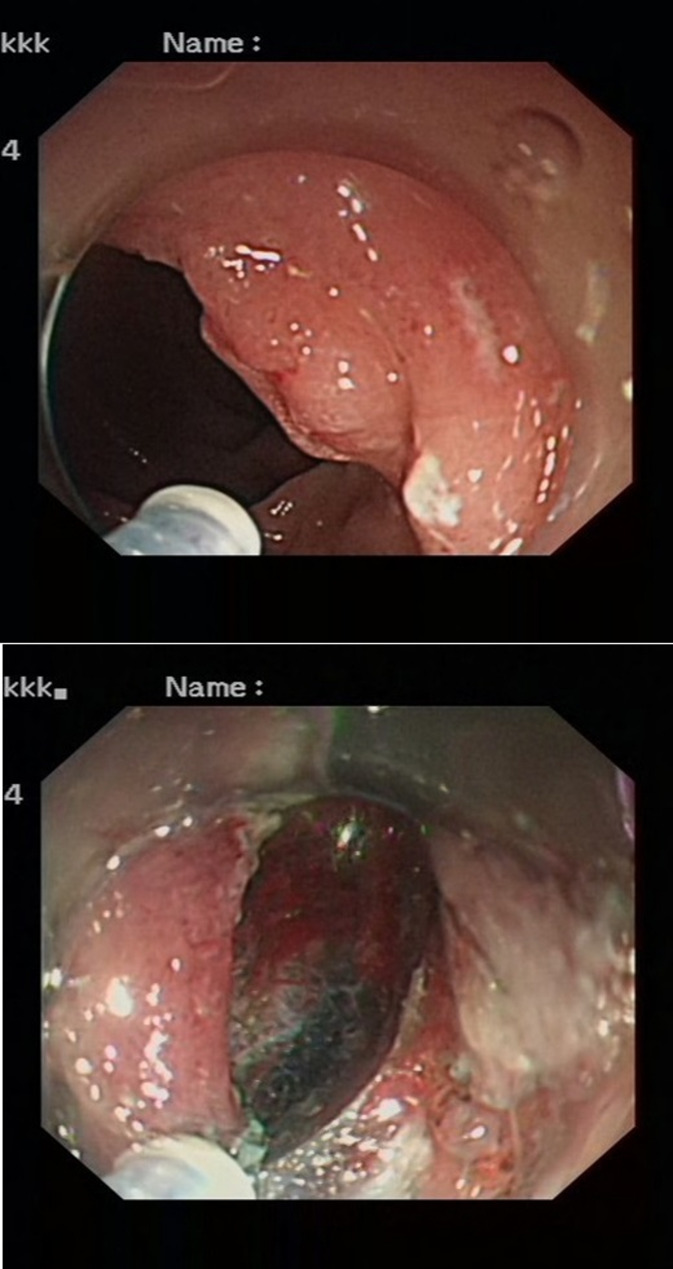
Endoscopic submucosal dissection of duodenal lesions

**Fig. 9:**
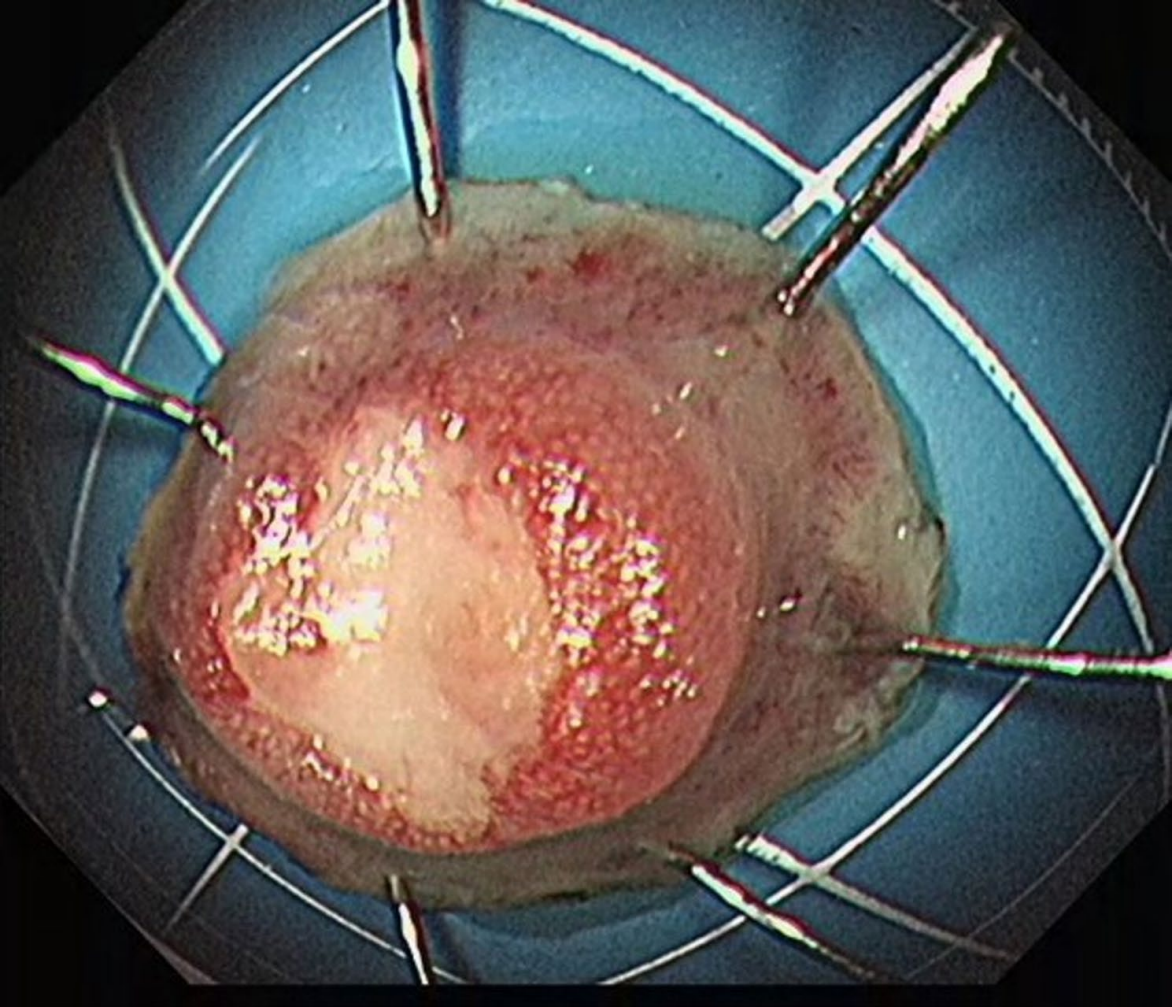
Endoscopic submucosal dissection of rectal lesions

In endoscopic mucosal resection, a transparent cap is preferred at the tip of a conventional upper gastrointestinal endoscope and a crescent-type snare. The snare was performed under submucosal injection of 1:10,000 saline: adrenaline for gastric (Fig. 4), duodenal (Fig. 5), and rectal lesions (Fig. 6). Similarly, endoscopic submucosal dissection was performed for gastric lesions (Fig. 7), duodenal lesions (Fig. 8), and rectal lesions (Fig. 9). A knife was used to make incisions. Subsequently, a complete incision was made. General anesthesia is preferred in surgeries. R0 resection was achieved after all the endoscopic surgical procedures. Endoscopic resection with R0 margins was achieved for no lymphovascular invasion, and submucosal invasion ≤ 500 μm. The other curative resection criteria were followed the European Society of Gastrointestinal Endoscopy (ESGE) guidelines [15]. The criteria for choosing endoscopic or surgical treatment at our hospital are endoscopic and surgeons’ decisions. When lesions 1.5–3 cm in diameter endoscopic mucosal resection was performed, and endoscopic submucosal dissection was performed when lesions of smaller size were observed. Endoscopic ultrasound (EUS) and/or biopsy with histological assessment) routinely performed on all lesions included in this study for lesion confirmation prior to endoscopic resection. The endoscopic resections reported represent the first treatment attempt for these lesions.

### Collections of data

Data regarding demographic and clinical parameters, endoscopic surgical procedure parameters, histopathological results, condition(s) after surgery, and follow-up data (including recurrences) were collected from the hospital records of patients and analyzed.

### Classification of surgical specimen of lesion

The WHO classification system was used to classify surgical specimens of gastric, duodenal, and rectal neoplasm lesions after endoscopic mucosal or submucosal dissection of neuroendocrine tumors [1].

### Peri-and post-procedural complications

Periprocedural complications occur during the endoscopic surgical procedure, and post-procedural complications occur after the endoscopic surgical procedure. Patients were followed 15 days after the endoscopic resection to define post-procedural complications.

### Bleeding

Hemostasis is required, and bleeding is considered during or after the endoscopic surgical procedure.

### Perforation

Bowel wall penetration was considered as a perforation. This was confirmed using computed tomography.

### Tumor size

Tumor size was in terms of pathologic size.

### Recurrences

Immunity Somatostatin receptor type 2 A in neuroendocrine tumor resection specimens by histochemical EnVision staining (SSTR2A, somatostatin receptor subtype 2 A), insulinoma-associated protein 1 (INSMl, Insulinoma associated-1), and synaptic vesicles (Syn, synaptophysin in), and the expression of chromopin A (CgA, chromogranin A), CD56, and Ki-67 were used to identify recurrences. In addition, a biopsy of a suspicious lesion was performed to identify recurrence, especially in cases of distant recurrence. Recurrences that occurred near the lesion were considered local recurrences. Recurrences that occurred in the liver and/or other organs were considered distal metastases. Pathological evaluation of the resected specimens was performed for this analysis. Cases of local recurrence characterized by positive margins.

**Fig. 10:**
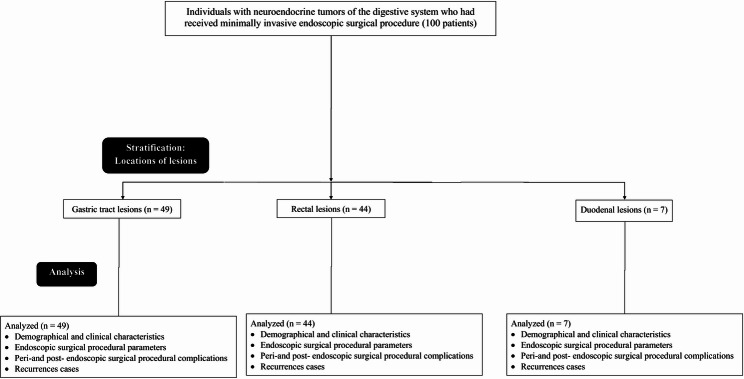
The retrospective study flow diagram (according to different locations of lesions)

The retrospective study flow diagram is presented in Fig. 10.

### Statistical analysis

InStat 3.01 GraphPad Software (San Diego, CA, USA) was used for the statistical analysis. Categorical, continuous normally distributed, and continuous non-normally distributed variables are presented as frequencies with percentages in parentheses, mean ± standard deviation (SD), and median with Q3–Q1 in parentheses. Quartile calculator, Calculator Soup^®^, LLB, USA (https://www.calculatorsoup.com/calculators/statistics/quartile-calculator.php) was used to calculate the quartile values. Kolmogorov and Smirnov test was used to check the normality of continuous variables. One-way analysis of variance (ANOVA) was used for continuous normally distributed variables. Bartlett’s test was used to test the homogeneity of normally distributed continuous variables. The Kruskal–Wallis’ test was used for statistical analysis of nonparametric ANOVA. Univariate following multivariable logistic regression analysis was performed to evaluate the association of the indicators (demographic and clinical characteristics, endoscopic surgical procedural parameters, peri-and post-endoscopic surgical procedural complications) with local and/or distal metastases in the follow-up period (odds ratio > 1 and *p*-value < 0.05, were considered significant parameters) [1]. The variables for the model were selected using a theoretical background. All results were considered significant at a 95% confidence interval (CI) and *p*-value less than 0.05.

## Results

### Patient characteristics

Male and female patients were equally distributed among those with gastric, duodenal, and rectal lesions. Most of the patients were Han Chinese. Patients generally have WHO classification of grade 1 or grade 2 lesions. A total of 49 (49%), 44 (44%), and 7 (7%) lesions were gastric, rectal, and duodenal lesions, respectively. Endoscopic mucosal resection was preferred for endoscopic surgery (63% of patients) and endoscopic submucosal dissection was performed in 37% of patients. Age ranged from 40 to 65 years for patients whose data were included in the analyses. There were no statistically significant differences in demographic and clinical characteristics according to the location of lesions (*p* > 0.05). Demographic and clinical characteristics of the enrolled patients are shown in Table 1.

**Table 1:** Demographical and clinical characteristics of enrolled patients

Parameters		-	Different locations of lesions			Comparisons among different locations of lesions		
		Total	Gastric tract lesions	Rectal lesions	Duodenal lesions			
Numbers of patients		100	49	44	7	*p*-value	Test value	Df
Gender	Male	58(58)	25(51)	29(66)	4(57)	0.348 (*χ*^2^-test for independence)	2.112	2
	Female	42(42)	24(49)	15(34)	3(43)			
Age (years)		52.79 ± 0.68	53.65 ± 6.92	51.66 ± 6.95	53.85 ± 4.67	0.342 (one-way ANOVA)	1.084	99
Ethnicity								
Han Chinese		91(91)	45(92)	40(91)	6(86)	0.943 (*χ*^2^-test for independence)	0.769	4
Mongolian		7(7)	3(6)	3(7)	1(14)			
Tibetan		2(2)	1(2)	1(2)	0(0)			
^#^Classification of surgical specimen of lesion								
Grade 1		60(60)	26(53)	29(66)	5(71)	0.367 (*χ*^2^-test for independence)	2.004	2
Grade 2		40(40)	23(47)	15(44)	2(29)			
Endoscopic dissection procedure								
Endoscopic mucosal resection		63(63)	31(63)	27(61)	5(71)	0.805 (*χ*^2^-test for independence)	0.434	2
Endoscopic submucosal dissection		37(37)	18(37)	12(39)	2(29)			
Associated disease								
Diabetes		7(7)	3(6)	3(7)	1(14)	0.705 (*χ*^2^-test for independence)	0.7	2
Hyperthyroidism		1(1)	1(2)	0(0)	0(0)	0.436 (*χ*^2^-test for independence)	1.658	2

Categorical and continuous normally distributed variables are depicted as frequencies with percentages in parenthesis and mean ± standard deviation (SD)

^#^WHO classification

df: degree of freedom, *χ*^2^-test: Chi-square test, ANOVA: Analysis of variance

Test value (*χ*^2^-value for *χ*^2^-test, F value for ANOVA)

### Endoscopic procedure characteristics

The tumor size ranged from 6 to 11.3 mm (minimum value to maximum value) and the endoscopic procedure time from 6 to 13 min (minimum value to maximum value) were reported (the reported procedure times for surgical procedure includes only the time for removal of lesions. The total time from the start of anesthesia to discharge from the operating theater to the postoperative intensive care unit was higher). A total of six (6%), four (4%), 16 (16%), and five (5%) patients reported bleeding, perforation, nausea, and vomiting, respectively, due to endoscopic surgical procedures. None of the patients had delayed bleeding, perforation, or stenosis. There were no statistically significant differences among endoscopic surgical procedure characteristics and peri-and post-endoscopic surgical procedural complications according to the location of lesions (*p* > 0.05). Details of the endoscopic surgical procedure characteristics of the enrolled patients, including complications, are presented in Table 2.

**Table 2:** Endoscopic surgical procedural characteristics of enrolled patients

Parameters	-	Different locations of lesions			Comparisons among different locations of lesions		
	Total	Gastric tract lesions	Rectal lesions	Duodenal lesions			
Numbers of patients	100	49	44	7	*p*-value	Test value	Df
Tumor size (mm)	9.3 ± 1.3	9.49 ± 1.28	9.02 ± 0.91	9.66 ± 0.96	0.090 (One way ANOVA)	2.468	99
Procedure time (min)	10(11.25–8.75)	10.5(11–9.5)	9.25(11.75–8)	11(11.5–9.5)	0.194 (Kruskal–Wallis’ test)	3.281	N/A
Peri-and post-procedural complications							
Bleeding	6(6)	2(4)	3(7)	1(14)	0.391 (Freeman-Halton extension of Fisher’s exact)	1.224	2
Perforation	4(4)	2(4)	1(2)	1(14)	0.344 (Freeman-Halton extension of Fisher’s exact)	2.271	2
Nausea	16(16)	10(20)	5(11)	1(14)	0.4898 (*χ*^2^-test for independence)	1.427	2
Vomiting	5(5)	4(8)	1(2)	0(0)	0.562 (Freeman-Halton extension of Fisher’s exact)	2.151	2

Categorical, continuous normally distributed variables, and continuous non- normally distributed variables are depicted as frequencies with percentages in parenthesis, mean ± standard deviation (SD), median (Q3–Q1)

df: degree of freedom, N/A: not applicable, ANOVA: Analysis of variance, Q3: Third quartile, Q3: First quartile

Test value (*χ*^2^-value for *χ*^2^-test, F value for ANOVA, Kruskal–Wallis’ statistics for Kruskal–Wallis’ test; relative risks for Fisher’s exact test)

### Prevalence of recurrence

**Fig. 11:**
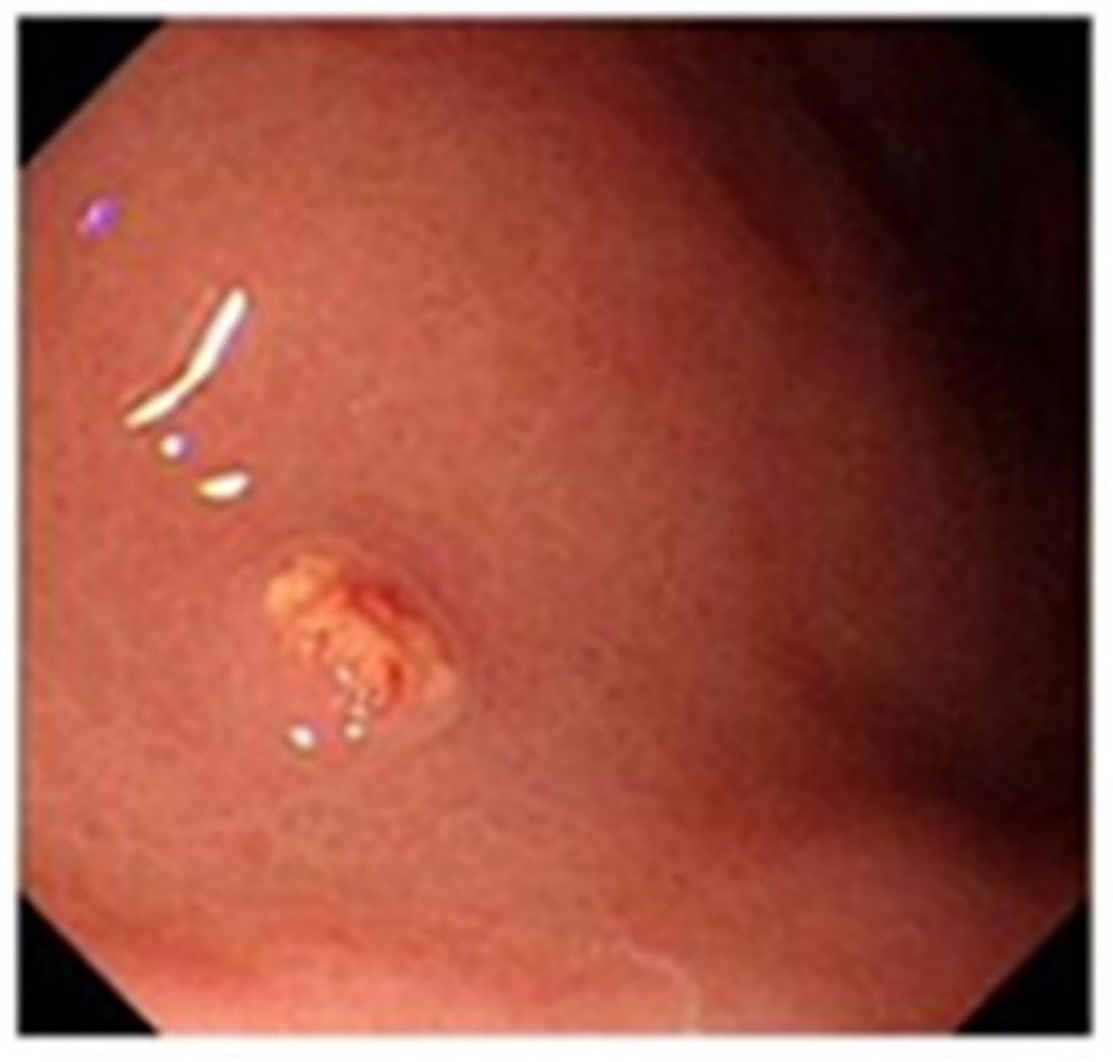
Case of gastric local recurrence

**Fig. 12:**
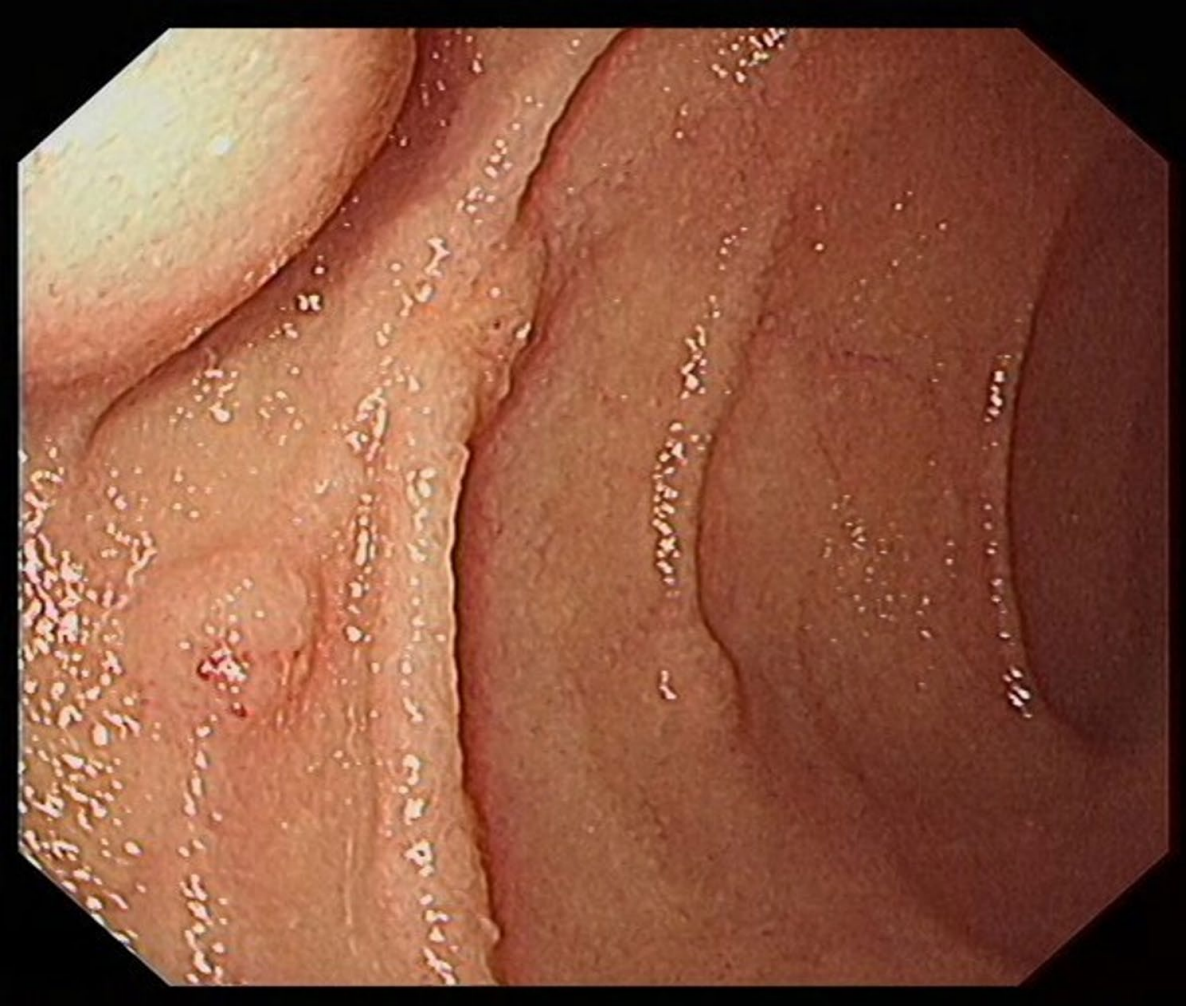
Case of duodenal local recurrence

None of the patients died during the follow-up period. Five (5%) patients experienced recurrence among the study patients. Local recurrences occurred in three (3%) patients, and distal metastases occurred in two (2%) patients. A total of two (2%) local recurrences occurred in the gastric lesions (Fig. 11), and one (1%) local recurrence occurred in duodenal lesions (Fig. 12). Rectal recurrence was not observed in the study population. All local recurrences were graded as one type (differentiation was good and the risk of metastasis was low for local recurrences). Distal metastases rates were also low. Generally, liver or extrahepatic metastases are observed. One patient had liver metastasis (WHO classification grade 2) and one patient had extrahepatic metastasis (WHO classification grade 2). Neuroendocrine tumors were not located in the stomach, duodenum, or rectum in patients with distal metastases. The details of the prevalence of recurrence are shown in Table 3.

**Table 3:** Cases of recurrences

Type of recurrences	Total	Location of recurrences	^#^Classification of surgical specimen of lesion
Local recurrences	3(3%)	Gastric recurrences: 2	Grade 1
		Duodenal recurrence: 1	Grade 1
Distal metastases	2(2%)	Liver: 1	Grade 2
		Vital organ other than liver: 1	Grade 2
Total (local recurrences or distal metastases)	5(5%)	local recurrences or distal metastases	Grade 1 or grade 2

^#^WHO classification

The cases with local recurrence characterized by positive margins

### Association of recurrence

Univariate analyses reported that age 65–53 years, male sex, Han Chinese ethnicity of patients, surgical specimen of lesion of grade 2, endoscopic mucosal resection surgical procedure, 11.3–9.5 mm tumor size, 13–10 mm procedure time, gastric tract and rectal lesions, and peri-and post-procedural complications were associated with local and/or distal metastases (*p* < 0.05; data not shown). Before surgery, grade 2 tumors, tumor size 9.5 mm or more, and gastric tract and rectal lesions were associated with local and/or distal metastases. The details of the indicators (demographic and clinical characteristics, endoscopic surgical procedural parameters, peri-and post-endoscopic surgical procedural complications) that were used in the study to evaluate the association with local and/or distal metastases are presented in Table 4 (local recurrence and distal metastases are mixed in the same analysis).

**Table 4:** Association of the indicators (demographical and clinical characteristics, endoscopic surgical procedural parameters, peri-and post- endoscopic surgical procedural complications) for local and/ or distal metastases

Parameters	Odd ratio	95%CI	*p*-value
Age (65–53 years vs. 53–40 years)	0.984	0.891–1.012	0.052
Gender (female vs. male)	0.084	0.074–1.021	0.082
Ethnicity (Han Chinese vs. Others)	0.521	0.042–0.084	0.124
Surgical specimen of lesion (Grade 2^*^vs. Grade 1)	1.214	1.121–1.451	0.049
Endoscopic surgical procedure (endoscopic mucosal resection vs. endoscopic submucosal dissection)	0.622	0.421–0.842	0.089
Tumor size (11.3–9.5^*^ mm vs. 6–9.5 mm)	1.542	1.222–2.421	0.041
Procedure time (13–10 min vs. 10–6 min)	0.421	0.322–0.654	0.241
Peri-and post-procedural complications (present vs. absent)	0.854	0.741–1.042	0.076
Locations of lesions (gastric tract and rectal lesions^*^ vs. duodenal lesions)	1.2411	1.1112–1.561	0.021

Logistic regression analyses

CI: Confidence interval

An odd ratio > 1 and *p*-value < 0.05 were considered significant parameters

^*^Significant parameter for recurrences

R0 resection was achieved after all endoscopic surgical procedures

The results of the assumption tests are presented in Table 5.

**Table 5:** Checking the assumptions of the models or statistical tests used

Variables	Test with *p*-value
Categorical variables	
2 × 2 tables; 2 × 3 tables	Fisher’s exact test or Chi-square test (*χ*^2^-test) with Yate’s corrections (for sample sizes of more than 5 and a total sample of more than 50).
Large tables	*χ*^2^-test for independence (when all expected values are greater than 1.0 and at least 20% of the expected values are greater than 5.) or Freeman-Halton extension of Fisher’s exact (only if *N* ≤ 300)
Continuous variables	
Demographical and clinical characteristics	
Age (years)	All columns passed the normality test (*p* > 0.1 for all) and the *p*-value for the Bartlett test was 0.5055 i.e. One-way Analysis of Variance (ANOVA)
Dissection procedure characteristics	
Tumor size (mm)	All columns passed the normality test (*p* > 0.1 for all) and the *p*-value for the Bartlett test was 0.0714, i.e. one-way ANOVA
Procedure time (min)	All columns passed the normality test (*p* > 0.1 for all) and the *p*-value for the Bartlett test was 0.0206, i.e. Kruskal–Wallis’ test
Multivariable logistic regression analysis	A linear relationship between predictors and the logit of the response variable. Therefore, Box–Tidwell test
Significant value	An odd ratio > 1 and *p*-value < 0.05 were considered significant parameters.

ANOVA: Analysis of variance; *χ*^2^-test: Chi-square test

## Discussions

The prevalence of local recurrences was only 3% and that of distal metastases was 2% during the follow-up period in the current study. The results of the local and distal metastases in the current study are consistent with those of single-center retrospective studies [1, 16], open-label, prospective studies [17], another retrospective study [18], case reports [19], and multicenter, retrospective studies [20, 21]. Endoscopic submucosal dissection or endoscopic mucosal resection of neuroendocrine tumors of the digestive system has fewer local recurrences and distal metastases. The details of demographic and clinical characteristics, endoscopic surgical procedure parameters, and recurrence cases of patients in comparative studies on endoscopic submucosal dissection or endoscopic mucosal resection of neuroendocrine tumors of the digestive system in different settings are presented in Table 6.

**Table 6:** The details of demographical and clinical characteristics, endoscopic surgical procedure parameters, and recurrence cases of patients in the comparative studies on endoscopic submucosal dissection or endoscopic mucosal resection of neuroendocrine tumors of the digestive system in different settings

Study	Published year	Study population	Sample size (*N*; patients)	Age (years)	Follow-up	Recurrences	Main conclusions
Single-center retrospective study, Pimentel-Nunes et al. [1]	2023	Portuguese	53	59(38–77)	44.6 months	4(8%)	Endoscopic resection is a safe and highly effective treatment, especially for luminal neuroendocrine tumors of the digestive tract < 12 mm.
Retrospective analysis, Suzuki et al. [16]	2012	Japanese	42	55 ± 14	37 months	0(0%)	Endoscopic submucosal dissection is a safe and effective endoscopic treatment for rectal and gastric carcinoid tumors.
Open-label, prospective study, Uygun et al. [17]	2014	Turkish	22	38–67	7 years (range: 2–14 years)	4(18%)	Endoscopic resection of type 1 gastric neuroendocrine tumors is a safe and effective treatment option with a relatively low recurrence rate.
Retrospective study, Jung et al. [18]	2015	Korean	49	> 18	Range: 3–10 years	5(10%)	The prognosis of patients with neuroendocrine tumors of the digestive tract treated by endoscopic resection may be good if the tumor size is small and histologically low grade without lymphatic invasion.
Case report, Matsumoto et al. [19]	2011	Japanese	5	69 ± 6	Range: 1–3 years	0(0%)	Endoscopic submucosal dissection is useful for treating gastric lesions that are ≤ 10 mm in diameter and show invasion to the submucosal level.
A multicenter, retrospective study, Kim et al. [20]	2014	Korean	41	63(38–79)	17 months (range 1–53 months)	0(0%)	Endoscopic submucosal dissection is useful for treating duodenal lesions that are ≤ 10 mm in diameter and show invasion to the submucosal level.
Retrospective analysis, Gupta et al. [21]	2023	Western	15	64(58–70)	19.9 months (range 10.3–49.3)	0(0%)	Experience is required to be considered for the endoscopic resection procedure.
Retrospective study, Kim et al. [22]	2022	Korean	427	53(46–63)	36(15–63) months	34(8%)	Neuroendocrine tumors of the digestive tract have a good prognosis.
Retrospective analysis, Kwon et al. [23]	2013	Korean	119	58.6(25–85)	5 years	0(0%)	Endoscopic resection procedure is not preferred for Grade 3 neuroendocrine tumors of the digestive system.

Variables are depicted as median with Q3–Q1 in parentheses

Patients with grade 3 tumors who successfully underwent endoscopic submucosal dissection or resection at our institute were excluded from the study. Grade 3 tumors are more complex and aggressive [24]. Therefore, the guidelines recommend open surgeries [5, 6]. The selection procedure for endoscopic submucosal dissection or endoscopic mucosal resection for grade 3 in the current study is consistent with those of a single-center retrospective study [1] and a retrospective analysis [23]. Endoscopic submucosal dissection or endoscopic mucosal resection can be used for neuroendocrine grade tumors, such as neuroendocrine grade 1 and 2 tumors of the digestive system. However, grade 3 neuroendocrine tumors of the digestive system grow rapidly and metastasize at an early stage, which is not an indication for endoscopic surgery [22, 25], and grade 3 neuroendocrine tumors of the digestive system should be removed from the evaluation criteria, and only grade 1 and grade 2 neuroendocrine tumors of the digestive system suitable for endoscopic resection were included in the study.

The prevalence and recurrence of duodenal lesions were lower than those of gastric lesions for neuroendocrine tumors in the digestive system. The results of the prevalence and recurrence of duodenal lesions in the current study are consistent with those of single-center retrospective studies [1, 16], case reports [19], and multicenter retrospective studies [20, 21]. Endoscopic submucosal dissection or endoscopic mucosal resection for duodenal lesions is similar to that for gastric lesions.

There were fewer periprocedural and postprocedural complications among the patients. In addition, peri-and post-procedural complications showed no significant differences in the locations of the lesions and were not associated with recurrence. The results of peri-and post-procedural complications in the current study are not consistent with those of case reports [19] and multicenter retrospective studies [20, 21], but are consistent with single-center retrospective studies [1, 16]. A small sample size (type I error) is the reason for the contradictory results with case reports [19] and multicenter retrospective studies [20, 21]. In addition, Grade 3–4 adverse events were more frequently reported if neuroendocrine tumors of the digestive system if they are treated using chemotherapy [26]. Peri-and post-procedural complications are manageable with endoscopic submucosal dissection or endoscopic mucosal resection of neuroendocrine tumors of the digestive system.

In the current study, endoscopic submucosal dissection or resection was successfully performed for rectal lesions with manageable adverse effects and no recurrence. The results of endoscopic submucosal dissection or endoscopic mucosal resection for rectal lesions in the current study are consistent with those of single-center retrospective studies [1] and meta-analyses [10, 11]. Endoscopic submucosal dissection or endoscopic mucosal resection for rectal lesions is similar to that for gastric and duodenal lesions.

Grade 2 and tumor sizes ≥ 9.5 mm were associated with systematic and local recurrences. The recurrences were consistent with those in a single-center retrospective study [1]. Patient profiles were associated with most recurrences. To overcome these recurrences, it is necessary to overcome the worse parameters of patients before performing the endoscopic procedure.

One study found that peri- and postprocedural complications were not associated with recurrence. However, the results of the association of peri- and post-procedural complications with recurrence are inconsistent with those of other studies. A small sample size (type I error) was the reason for these contradictory results.

This study discussed the prevalence, recurrence, and metastasis of gastric, duodenal, and rectal neuroendocrine tumors after endoscopic resection. However, in the current study, none of the rectal recurrences were included in the analysis. Gastric and duodenal neuroendocrine tumors belong to the foregut, whereas rectal neuroendocrine tumors belong to the hindgut. Therefore, pathogenesis and behavior, which may be associated with metastasis, are different. Therefore, the analyses limited their study to gastric and duodenal recurrence after endoscopic resection.

This study has a clear structure with well-defined sections (abstract, introduction, methods, results, and discussion). This retrospective design allows efficient data collection from a specialized center. However, the study has limitations, such as the small sample size and fewer follow-up periods. The stratification of variables was based on the location of lesions, but not according to the type of surgical procedure (endoscopic submucosal dissection or endoscopic mucosal resection). A dynamic study with long-term follow-up is required. This study did not consider endoscopists who performed endoscopic treatments. Complication rates for endoscopic procedures differ depending on whether they are performed by trainees or experts. Gastric neuroendocrine tumors are also classically categorized into three types according to their etiology, known as the Rindi Classification of Endocrine Cell Neoplasms. The rate of lymph node metastasis differs according to type. When the study considered the recurrence and metastasis of gastric neuroendocrine tumors, the Rindi classification, which may be a confounding factor, should also be mentioned. However, the current study did not report on the Rindi classification. After pathological diagnosis of gastric and rectal endocrine tumors, venous invasion is reported to be a risk factor for lymph node metastasis. When the study considers recurrence and metastasis of gastric and rectal neuroendocrine tumors, the pathological diagnosis, including venous invasion, should also be mentioned. A possible justification for this finding is that the study did not report any lymph node metastasis. Some guidelines have a cutoff of 1–2 cm. However, in the current study, the tumor diameter was set as the risk factor for tumor recurrence and metastasis at 9.5 mm. References of previous studies and institutional instructions are the reasons for the 9.5 mm was set as threshold of the tumor diameter and the risk of tumor recurrence and metastasis in this study. Only seven patients were included in the duodenal subgroup, has only 7 patients, which limits the statistical power and generalizability. Subgroup analyses (e.g., recurrence by location) may be unreliable. The possible justifications for the same are that duodenal lesions are always fewer in number. This study focused on the Chinese population. This is a limitation to the generalizability of the findings. Future research, such as conducting multicenter studies with diverse populations, would strongly support this hypothesis.

## Conclusions

The prevalence of neuroendocrine tumors is high in the stomach and rectum, and rare in the duodenum. Endoscopic submucosal dissection or endoscopic mucosal resection of neuroendocrine tumors of the digestive system has few local and distal metastases. Endoscopic submucosal dissection or endoscopic mucosal resection may be used for grade 1 and 2 neuroendocrine tumors of the digestive system. Endoscopic submucosal dissection or endoscopic mucosal resection for gastric, duodenal, and rectal lesions is effective with manageable peri-and post-procedural complications, especially for grade 1 and less than 9.5 mm tumor sizes. Before surgery, grade 2 and tumor sizes 9.5 mm or more were independent parameters for systematic and local recurrences after endoscopic submucosal dissection or endoscopic mucosal resection of neuroendocrine tumors of the digestive system. This study addressed an important clinical question regarding the safety and recurrence risks of endoscopic resection for neuroendocrine tumors in the digestive system. However, the findings may be limited to a specific setting. Operator experience and tumor biology should be considered potential confounding factors.

## Supplementary Information

Below is the link to the electronic supplementary material.

Supplementary Material 1

## Data Availability

The datasets used and analyzed during the current study are available from the corresponding author upon reasonable request.

## Declarations

### Declaration of figures authenticity

All figures submitted were created by the authors (or collected from the medical records), who confirmed that the images were original with no duplication and have not been previously published in whole or in part.

All the data and related metadata underlying the reported findings have already been provided as part of the submitted article. There are no supplementary files (supplementary tables, supplementary figures, and others) referred to in this manuscript. Therefore, there is nothing to deposit in the appropriate public data repositories.

## Abbreviations

SD: Standard deviations
χ^2^-test: Chi-square test
ANOVA: Analysis of variance
CI: Confidence interval
STROBE: The reporting of this study conforms to the Strengthening the Reporting of Observational Studies in Epidemiology
