# Supplementary material for: Complications and recurrence risks after endoscopic resection of digestive neuroendocrine tumors: a retrospective study
Source: Orphanet J Rare Dis. 2025 Sep 2;20:472. doi: 10.1186/s13023-025-03992-x (PMC12403926; doi:10.1186/s13023-025-03992-x)
Supplement: Supplementary file 1 — Supplementary Material 1 [file 13023_2025_3992_MOESM1_ESM.pdf]

Stratification: Locations of lesions

In retrospective study, individuals with neuroendocrine tumors of the digestive system who had received minimally invasive endoscopic surgical procedure (100 patients)

Endoscopic ultrasound (EUS) and/or biopsy with histological assessment) routinely performed on all lesions included in this study for lesion confirmation prior to endoscopic resection.

The curative resection criteria were followed the European Society of Gastrointestinal Endoscopy (ESGE) guidelines

Gastric tract lesions (n = 49)

Rectal lesions (n = 44)

Duodenal lesions (n = 7)

WHO classification system was used to classify surgical specimens

None of the patients died in the follow-up period.

- Six (6 %), 4 (4 %), 16 (16 %), and 5 (5 %) patients reported bleeding, perforation, nausea, and vomiting, respectively due to surgical procedure(s).

- Five (5 %) patients suffered from recurrent endoscopic surgeries.
- Local recurrences: 3 (3 %; 2 (2 %) in gastric lesions and 1 (1 %) in duodenal lesions; all grade 1) patients
- Distal metastases: 2 (2 %) patients.

Before surgery, grade 2 ($p = 0.049$), tumor size ≥ 9.5 mm ($p = 0.041$), and gastric tract and rectal lesions ($p = 0.021$) were associated with local and/or distal metastases.

The prevalence of neuroendocrine tumors is high in the stomach and rectum, endoscopic resection may be safe, and high-grade tumors may be associated with a high risk of recurrence.
